# A Nano-sized Supramolecule Beyond the Fullerene Topology**

**DOI:** 10.1002/anie.201407120

**Published:** 2014-10-06

**Authors:** Fabian Dielmann, Claudia Heindl, Florian Hastreiter, Eugenia V Peresypkina, Alexander V Virovets, Ruth M Gschwind, Manfred Scheer

**Affiliations:** Institut für Anorganische Chemie, Universität Regensburg93040 Regensburg (Germany); Institut für Organische Chemie, Universität Regensburg93040 Regensburg (Germany); Nikolaev Institute of Inorganic Chemistry, Siberian Division of RAS AcadLavrentyev str. 3, 630090 Novosibirsk (Russia); Novosibirsk State UniversityPirogova str. 2, 630090 Novosibirsk (Russia)

**Keywords:** copper, Cp ligands, phosphorus, self-assembly, supramolecular chemistry

## Abstract

The reaction of [Cp^Bn^Fe(η^5^-P_5_)] (**1**) (Cp^Bn^=η^5^-C_5_(CH_2_Ph)_5_) with CuI selectively yields a novel spherical supramolecule (CH_2_Cl_2_)_3.4_@[(Cp^Bn^FeP_5_)_12_{CuI}_54_(MeCN)_1.46_] (**2**) showing a linkage of the scaffold atoms which is beyond the Fullerene topology. Its extended CuI framework reveals an outer diameter of 3.7 nm—a size that has not been reached before using five-fold symmetric building blocks. Furthermore, **2** shows a remarkable solubility in CH_2_Cl_2_, and NMR spectroscopy reveals that the scaffold of the supramolecule remains intact in solution. In addition, a novel 2D polymer [{Cp^Bn^Fe(η^5^-P_5_)}_2_{Cu_6_(μ-I)_2_(μ_3_-I)_4_}]_*n*_ (**3**) with an uncommon structural motif was isolated. Its formation can be avoided by using a large excess of CuI in the reaction with **1**.

The chemistry of supramolecular aggregates is one of the most interesting and fascinating fields in current research.[1] Based on self-assembly, the formation of discrete nano-sized supramolecules is enabled.[2] In contrast to weak interactions, which mostly are non-directional, the formation of ligand–metal dative bonds allows the rational design of novel structural motifs.[3] Special attention has been paid to the design of spherical containers with defined inner cavities.[4] Recently, we have shown that the pentaphosphaferrocene [Cp*Fe(η^5^-P_5_)] (**1 a**) (Cp*=η^5^-C_5_Me_5_) acts with CuX (X=Cl, Br) as a building block for the formation of spherical supramolecules with the I_*h*_-C_80_ fullerene-like topology consisting of 12 five-membered rings and 30 six-membered units (Figure 1, left).[5a,b] However, their synthesis is accompanied by polymeric products and special synthetic conditions have to be applied to avoid these products.[5c–f] In addition, most of the products are barely soluble in common solvents. Also a series of 90-vertex balls has been isolated which have a slightly better solubility (Figure 1, right).[5c–f] It is of note that almost all attempts to obtain spherical supramolecules from CuI and pentaphosphaferrocene failed to date.[6]

**Figure 1:**
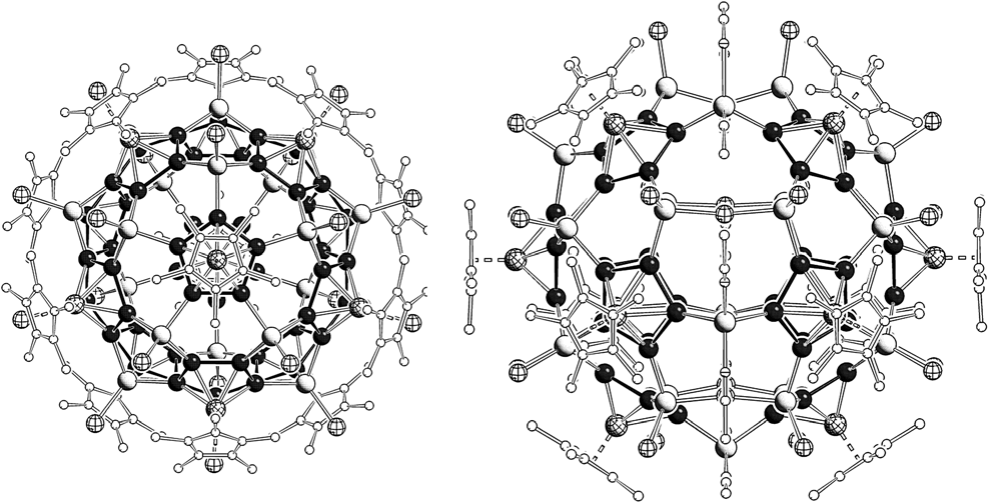
Examples of spherical supramolecules with fullerene-like topology self-assembled by 1 a and CuX (X=Cl, Br; incorporated templates are not shown). Left: 80-vertex ball, right: 90-vertex ball.

Herein we report the synthesis and characterization of a nano-sized spherical supramolecule, obtained by the self-assembly of [Cp^Bn^Fe(η^5^-P_5_)] (**1**) (Cp^Bn^=η^5^-C_5_(CH_2_Ph)_5_) and CuI, which shows a structure beyond the fullerene topology. Going from methyl to benzyl substituents at the Cp^R^ ligand, the steric influence of the ligand to the metal center remains similar,[7] but the formation of an even larger spherical molecule is achieved for the first time. This compound does not show a fullerene topology despite having 12 five-membered rings derived from pentaphosphaferrocenes because there are no six-membered rings in the spherical scaffold.[8] In addition, the flexible organic groups of the Cp^Bn^ ligand provide a good solubility of the resulting compound.

Layering a solution of CuI in a mixture of CH_2_Cl_2_ and MeCN over a solution of **1** in CH_2_Cl_2_ leads to the formation of (CH_2_Cl_2_)_3.4_@[{Cp^Bn^Fe(η^5^-P_5_)}_12_(CuI)_54_(MeCN)_1.46_] (**2**) isolated in good yields (69 %) [Equation (1)].

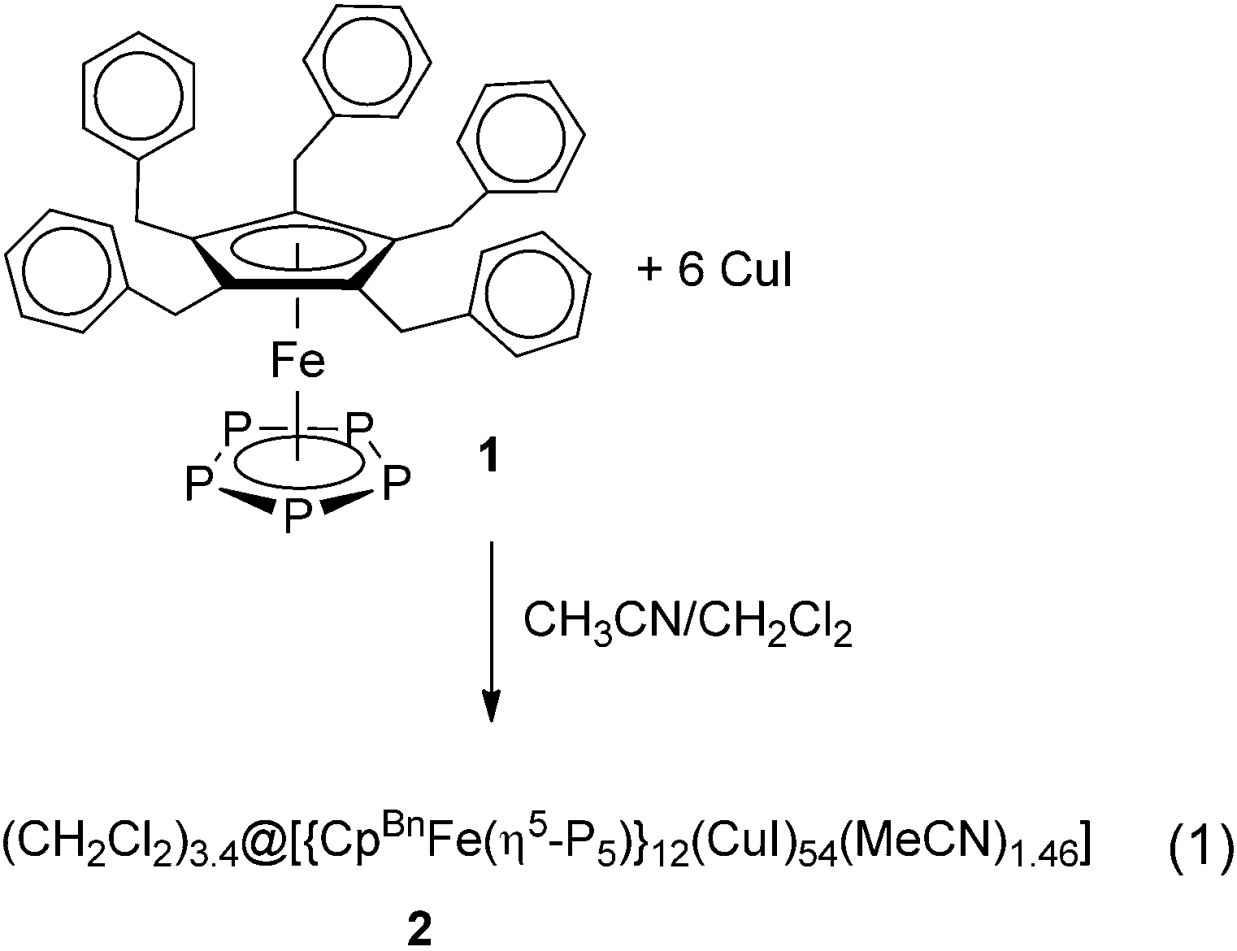


Unusually for this class of compounds **2** is soluble in CH_2_Cl_2_. Thus, NMR spectroscopic and mass spectrometric investigations have been carried out. The ^1^H NMR spectrum shows broad signals for both the phenyl H atoms (δ=7.0–6.0 ppm) and the methylene protons (δ=5.0–3.2 ppm) with an intensity ratio of 5:2. Weak signals in the range from δ=1.36 to 1.24 ppm can be assigned to coordinated acetonitrile ligands. The ^31^P{^1^H} NMR of **2** displays a broad signal at δ=77.3 ppm (*ω*_1/2_=630 Hz), which is in a comparable region to those found for 90-vertex supramolecules containing **1 a**[5c] and is indicative for a 1,2,3,4,5-coordination mode of the *cyclo*-P_5_ ligand to the Cu atoms. There is no signal found at 162 ppm for uncoordinated **1**. Thus, the scaffold of the giant molecule **2** remains intact in solution, which was demonstrated by diffusion ordered spectroscopy (DOSY) experiments. The DOSY-NMR experiment reveals a hydrodynamic radius of 2.07 nm, which is in very good agreement with the crystal-derived radius of 1.85 nm in the solid state.

The ESI mass spectrum displays the cations {[Cp^Bn^Fe(η^5^-P_5_)]_2_Cu_3_I_2_}^+^ and [Cu_9_I_8_]^+^ as the largest phosphorus-containing and phosphorus-free fragments, respectively, and {[Cp^Bn^Fe(η^5^-P_5_)]_2_Cu}^+^ as the base peak. In addition, several anionic fragments, indicative for the existence of large CuI frameworks, were detected: [Cu_14_I_15_]^−^ is the largest one and under subsequent elimination of CuI units all of the subsequent fragments down to [CuI_2_]^−^. Even in the MALDI mass spectrum only fragments of **2** with the cation {[Cp^Bn^Fe(η^5^-P_5_)]_2_Cu_2_I}^+^ as the largest mass peak and again {[Cp^Bn^Fe(η^5^-P_5_)]_2_Cu}^+^ as the base peak are detected.

Compound **2** crystallizes as deep red–brown blocks in the triclinic space group *P*$\bar 1$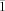 The supramolecule occupies the center of symmetry.[9] Its idealized scaffold can be represented as a combination of eight {CuI_4_} tetrahedral units and six similar building blocks, (Cp^Bn^FeP_5_)_2_(CuL)(Cu_2_I)_4_, where L=I in two and L=MeCN in four ones (Figure 2 a). The core of the L=MeCN building block comprises two molecules of **1** joined by one {CuL} and two {Cu_2_I} bridging units. The {CuL} unit is the only similarity to earlier reported 80-vertex supramolecules based on **1 a**,[5] where each CuX (X=Cl, Br) unit coordinates three molecules of **1 a**. In addition, each *cyclo*-P_5_ ligand of **1** is coordinated by two chelate {Cu_2_I} units. Therefore, each phosphorus atom is available for coordination to copper ions allowing an 1,2,3,4,5-coordination mode as in other pentaphosphaferrocene-based supramolecules.[5] Each of the (Cp^Bn^FeP_5_)_2_(CuL)(Cu_2_I)_4_ building blocks is connected to four {CuI_4_} tetrahedra and four other similar building blocks, which are rotated by 90° in two perpendicular directions (Figure 2 a). The building blocks are thus aggregated in an extended {CuI}_56_ ladder-like framework by formation of Cu=I bonds (see Supporting Information), constructed of eight {CuI_4_} tetrahedra and 12 {Cu_4_I_2_} units, that can be found in some CuX frameworks.[10] The eight {CuI_4_} tetrahedra are arranged according to the corners of a giant “cube” with six (Cp^Bn^FeP_5_)_2_(CuL)(Cu_2_I)_4_ building blocks as the folded convex faces. The CuI-rich idealized scaffold constructed in this way has the formula [(Cp^Bn^FeP_5_)_12_Cu_62_I_58_(MeCN)_4_] and must be positively charged owing to the excess of copper ions (Figure 2 c). However, according to the diffraction data for the single crystals of **2**, an average composition of (CH_2_Cl_2_)_3.4_@[(Cp^Bn^FeP_5_)_12_(CuI)_54_(MeCN)_1.46_]⋅2.54 MeCN⋅0.8 C_7_H_8_ is obtained. It is charge-balanced because some copper, iodine, and coordinated acetonitrile positions are statistically vacant. The vacant positions are distributed such that each copper atom retains its tetrahedral environment and each iodide is two-, three-, or four-fold coordinated. The idealized ladder-like framework {CuI}_56_ is thus reduced to its ordered part comprising of 34 copper and 40 iodine ions (Figure 2 d) with Cu=I bonds being in the range of 2.577(4)–2.800(3) Å. 20 copper and 14 iodide ions statistically decorate the ordered part (see Supporting Information). The vacancies in some of copper-ion positions can vary the coordination mode of the *cyclo*-P_5_ rings from the 1,2 and 1,2,3 found in the ordered {Cu_34_I_40_} part to the 1,2,3,4,5 mode that is achieved in the idealized structure. The Cu=P bonds vary in the range of 2.109(17) to 2.335(18) Å. The bonds Fe=P (2.345(8)–2.404(7) Å) and P=P (2.071(10)–2.123(11) Å) are comparable to 2.37 and 2.11 Å, respectively, in the non-coordinated pentaphosphaferrocene.[7]

**Figure 2:**
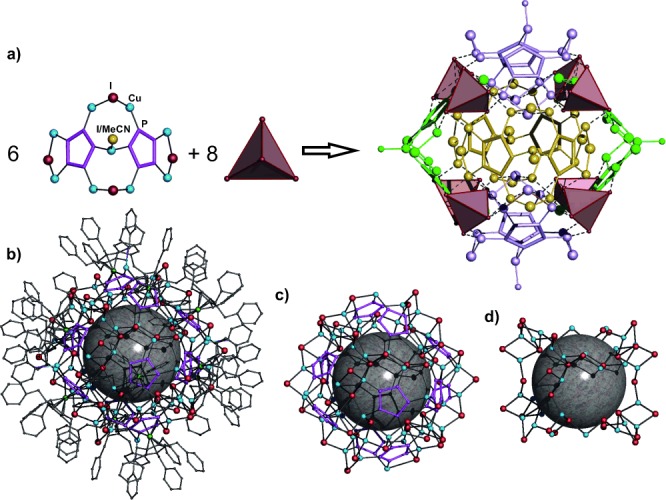
a) A combination of six (Cp^Bn^FeP_5_)_2_(CuX)(Cu_2_I)_4_ (X=I, MeCN) building blocks and eight CuI_4_ tetrahedra gives the idealized scaffold (Cp^Bn^FeP_5_)_12_Cu_62_I_58_(MeCN)_4_. b) One of the supramolecules in 2. Hydrogen atoms are omitted for clarity. c) The idealized scaffold of 2. d) The irreducible scaffold of 2.

Therefore, the crystal of **2** represents a solid solution of different similar supramolecules with the identical structural core comprising an ordered {Cu_34_I_40_} framework that predetermines the mutual spatial arrangement of 12 molecules of **1** as well as the shape of the supramolecule with an external size of 3.70 nm (Figure 2 b). They are the largest of the supramolecules built up by P_*n*_ ligand complexes, being by 1.2 nm, 0.3 nm, and 0.64 nm, respectively, larger than [Cp*Fe(η^5^-P_5_)]-containing spherical clusters.[5c,d], [6] For a more vivid comparison, the supramolecules in **2** are approximately 3.5 times larger in volume than the Buckminster fullerene C_60_. Even without the organic ligands, it is larger than the largest anionic copper(I) halide aggregate [Cu_36_I_56_]^20−^ reported to date.[11]

The supramolecules **2** have internal cavities of 0.75 nm, which is only 0.05 nm less in comparison to the 80-vertex supramolecule with **1 a** as building block.[5b] The cavities incorporate an average of 3.4 molecules of CH_2_Cl_2_ disordered over six possible positions (see Supporting Information). The surface of the inner cavities is formed by six idealized (Cp^Bn^FeP_5_)_2_(CuL)(Cu_2_I)_4_ building blocks that have folded convex geometry. Every guest molecule can occupy one of these six positions in the cavity oriented so that the Cl atoms point towards every idealized (Cp^Bn^FeP_5_)_2_(CuL)(Cu_2_I)_4_ building block according to the ‘concave to convex’ principle (see Supporting Information). The intermolecular distances between the P atoms of the *cyclo*-P_5_ rings and the Cl atoms of the guest molecule are about 3.8–3.9 Å, which correspond to weak van der Waals interactions.

The supramolecule **2** contains 4.5 CuI units per pentaphosphaferrocene. When the reaction is carried out with three or less equivalents of CuI, the polymeric by-product [{Cp^Bn^Fe(*η*^5^-P_5_)}_2_{Cu_6_(μ-I)_2_(μ_3_-I)_4_}]_*n*_ (**3**) can occasionally be observed [Equation (2)]. In contrast to **1 a** as building block, the tendency for the formation of polymeric products is much lower for **1** and can completely be avoided by using a higher amount of CuI.

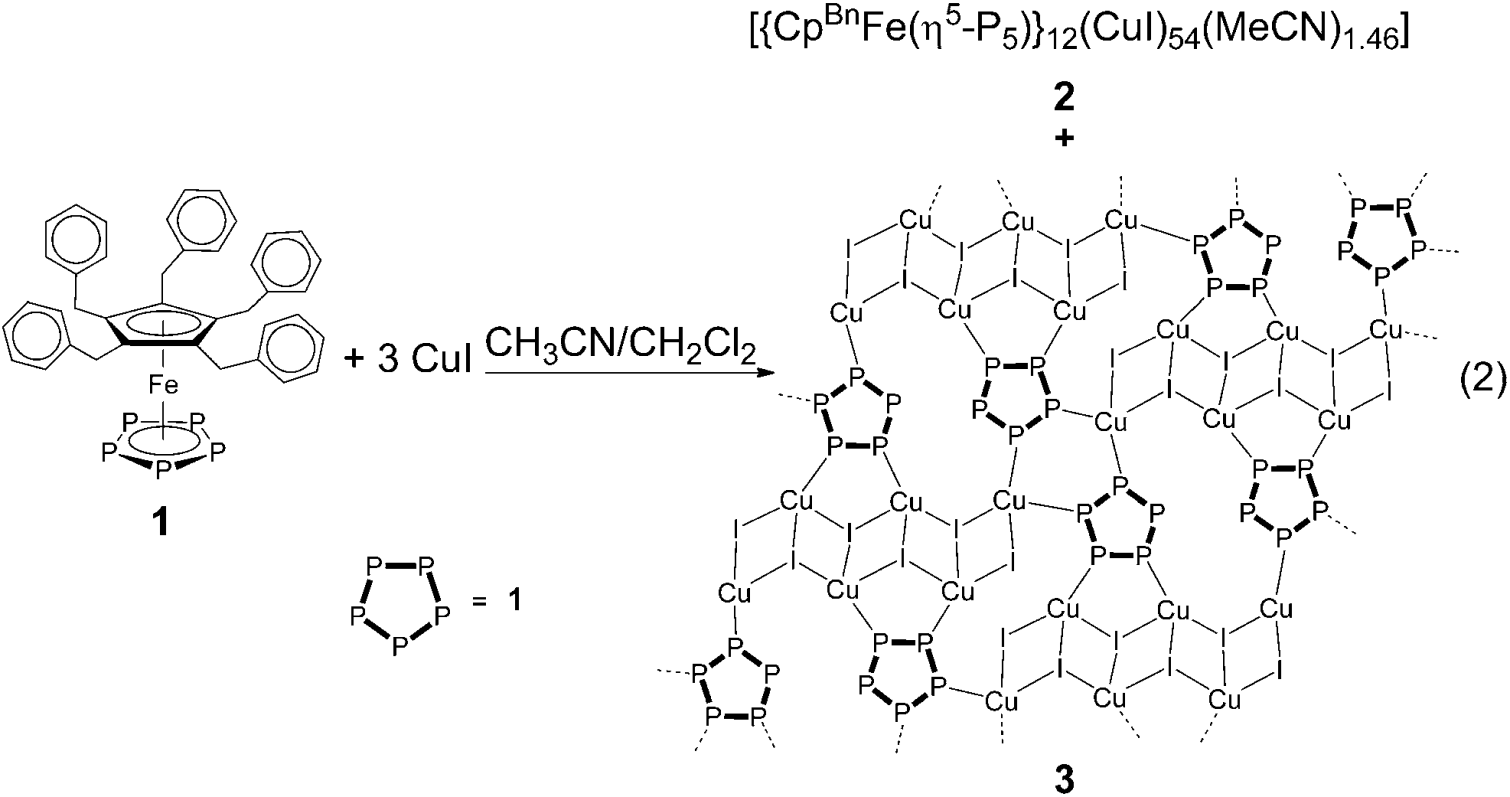


The 2D coordination polymer **3** crystallizes as yellow–orange plates in the triclinic space group *P*$\bar 1$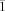.[9] It can easily be separated from the deep-red blocks of **2** either mechanically under the microscope or by washing the mixture with CH_2_Cl_2_, since **3** is insoluble in all common solvents. Single crystals of **3** were obtained from CH_2_Cl_2_/MeCN mixtures as solvate with one molecule CH_2_Cl_2_ per repeating unit. The structure of **3** is built up by planar layers of building block **1**, which binds to Cu_6_I_6_ units in the rather uncommon 1,2,3,4-coordination mode.[12] The framework therefore comprises *cyclo*-P_5_ ligands from the pentaphosphaferrocene, four-membered Cu_2_I_2_ rings of the ladder, five-membered Cu_2_P_2_I rings, and six-membered Cu_2_P_4_ rings (Figure 3). The unit of **1** alternates up and down respective to the layer because of the bulky benzyl substituents. Hence, the layers are faced by the phenyl rings of Cp^Bn^. A coordination polymer with **1 a** as building block and the same elemental formula was obtained earlier.[12] However, there the Cu_6_I_6_ units are not arranged in a ladder, but in six-membered rings instead. Hence, this demonstrates again the versatility of CuI in supramolecular and coordination chemistry.

**Figure 3:**
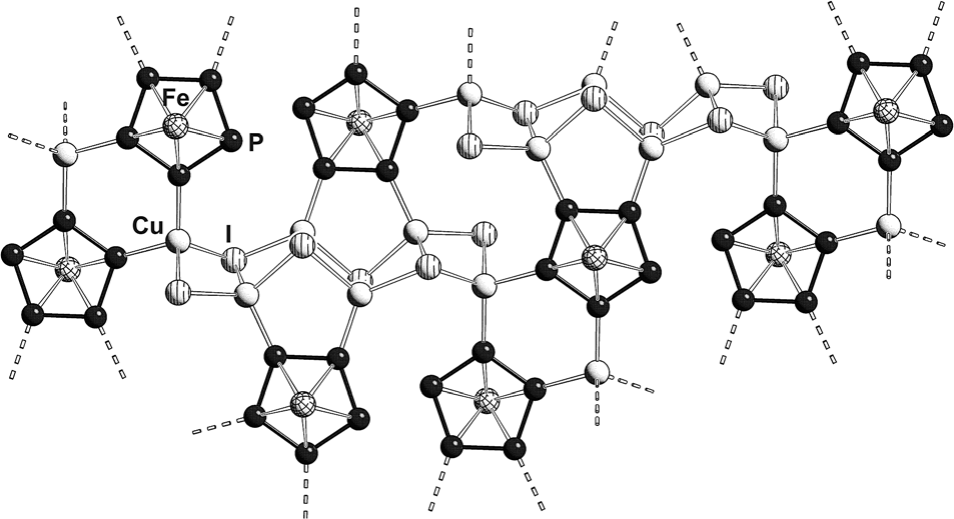
Section of the polymeric network of 3. Cp^Bn^ ligands are omitted for clarity. Selected bond lengths [Å]: Cu-I 2.5764(14)–2.7920(17), Cu-P 2.262(3)–2.301(3), P-P 2.099(3)–2.117(3) and Fe-P 2.363(3)–2.426(3).

In conclusion, the Cp^Bn^-substituted pentaphosphaferrocene **1** has proven to be a building block for unique spherical supramolecular aggregates. The replacement of Cp* by Cp^Bn^ can reverse the tendency for the formation of polymeric instead of spherical coordination compounds. In addition, the benzyl ligands provide unique solubility of the products that allowed the use of NMR and MS investigations. While polymeric products are favored in the case of **1 a**, the reaction of **1** and six equivalents of CuI selectively leads to the formation of a gigantic supramolecule **2** featuring an unprecedented scaffold. The inorganic scaffold consists of *cyclo*-P_5_ units and an expanded CuI framework with partial occupancies of few copper and iodine positions. It does not follow the fullerene-topology, because the CuI ladder structural motif provides no six-membered units, although twelve *cyclo*-P_5_ rings are present. Having 180 heavy atoms in the scaffold and an outer diameter of 3.70 nm, **2** represents the largest discrete polynuclear complex built up either by five-fold symmetric building blocks or copper(I) halide aggregates.

In memory of Reinhard Schmutzler

## References

[1a] Stupp SI, Palmer LC (2014). Chem. Mater.

[1b] Lanigan N, Wang X (2013). Chem. Commun.

[1c] Saalfrank RW, Scheurer A (2012). Top. Curr. Chem.

[1d] Dalgarno SJ (2010). Annu. Rep. Prog. Chem. Sect. B.

[1e] Mastalerz M (2010). Angew. Chem. Int. Ed.

[1f] Rehm TH, Schmuck C (2010). Chem. Soc. Rev.

[1g] Lehn J-M (2002). Proc. Natl. Acad. Sci. USA.

[1h] Cotton FA, Lin C, Murillo CA (2001). Acc. Chem. Res.

[1i] Bradley J, Holliday BJ, Mirkin CA (2001). Angew. Chem. Int. Ed.

[2] Lehn J-M (1988). Angew. Chem. Int. Ed. Engl.

[3a] Lindoy LF, Park K-M, Lee SS (2013). Chem. Soc. Rev.

[3b] Seidel SR, Stang PJ (2002). Acc. Chem. Res.

[4a] Young NJ, Hay BP (2013). Chem. Commun.

[4b] see Ref. [1 c];

[4c] Meng W, Clegg JK, Nitschke JR (2012). Angew. Chem. Int. Ed.

[4d] Laughrey Z, Gibb BC (2011). Chem. Soc. Rev.

[4e] Inokuma Y, Kawano M, Fujita M (2011). Nat. Chem.

[4f] Jin P, Dalgarno SJ, Atwood JL (2010). Coord. Chem. Rev.

[4g] Dalgarno SJ, Power NP, Atwood JL (2008). Coord. Chem. Rev.

[4h] Koblenz TS, Wassenaar J, Reek JNH (2008). Chem. Soc. Rev.

[4i] Schmuck C (2007). Angew. Chem. Int. Ed.

[4j] Heinz T, Rudkevich DM, Rebek J (1998). Nature.

[5a] Schindler A, Heindl C, Balazs G, Groeger C, Virovets AV, Peresypkina EV, Scheer M (2012). Chem. Eur. J.

[5b] Scheer M, Schindler A, Gröger C, Virovets AV, Peresypkina EV (2009). Angew. Chem. Int. Ed.

[5c] Scheer M, Schindler A, Bai J, Johnson BP, Merkle R, Winter R, Virovets AV, Peresypkina EV, Blatov VA, Sierka M, Eckert H (2010). Chem. Eur. J.

[5d] Welsch S, Groeger C, Sierka M, Scheer M (2011). Angew. Chem. Int. Ed.

[5e] Bai J, Virovets AV, Scheer M (2003). Science.

[5f] Scheer M, Bai J, Johnson BP, Merkle R, Virovets AV, Anson CE (2005). Eur. J. Inorg. Chem.

[6] Schwarzmaier C, Schindler A, Heindl C, Scheuermayer S, Peresypkina EV, Virovets AV, Neumeier M, Gschwind R, Scheer M, For the only existing example of a small baseball-like aggregate^[8]^ (2013). Angew. Chem. Int. Ed.

[7] Dielmann F, Merkle R, Heinl S, Scheer M (2009). Z. Naturforsch. B.

[8] In addition to **2**^[6]^*n*

[9] Crystal data for **2**_491.40__438.80__4__60__6.80__12__54__54_*P*
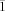
*a**b**c**α**β**γ**V*^3^*Z**D*_calcd_^−3^_Kα_*μ*^−1^^2^*R**I**wR*_2_*GooF*^−3^**3**_40.5__36__5__3__3_*P*
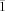
*a**b**c**α**β**γ**V**Z**D*_calcd_^−3^_Kα_*μ*^−1^^2^*R**I**wR*_2_*GooF*^−3^**2****3** http://www.ccdc.cam.ac.uk/data_request/cif.

[10] Peng R, Li M, Li D (2010). Coord. Chem. Rev.

[11] Hartl H, Fuchs J (1986). Angew. Chem. Int. Ed. Engl.

[12] Dielmann F, Schindler A, Scheuermayer S, Bai J, Merkle R, Zabel M, Virovets AV, Peresypkina EV, Brunklaus G, Eckert H, Scheer M (2012). Chem. Eur. J.

