# Supplementary material for: A Nano-sized Supramolecule Beyond the Fullerene Topology
Source: Angew Chem Int Ed Engl. 2014 Oct 6;53(49):13605–8. doi: 10.1002/anie.201407120 (PMC4501309; doi:10.1002/anie.201407120)
Supplement: Supplementary file 1 [file anie0053-13605-sd1.pdf]

Angewandte Chemie
Eine Zeitschrift der Gesellschaft Deutscher Chemiker

Supporting Information

© Wiley-VCH 2014

69451 Weinheim, Germany

A Nano-sized Supramolecule Beyond the Fullerene Topology**

*Fabian Dielmann, Claudia Heindl, Florian Hastreiter, Eugenia V. Peresypkina, Alexander V. Virovets, Ruth M. Gschwind, and Manfred Scheer**

anie_201407120_sm_miscellaneous_information.pdf

SUPPORTING INFORMATION

1. EXPERIMENTAL PART 2

1.1 Synthesis of $(CH_2Cl_2)_{3.4}$@[{$Cp^{Bn}Fe(\mu_6$-$\eta^5:\eta^1:\eta^1:\eta^1:\eta^1:\eta^1$-$P_5)$}$_{12}(CuI)_{54}(CH_3CN)_{1.46}$] (2) 2

1.2 Synthesis of [{$Cp^{Bn}Fe(\mu_5$-$\eta^5:\eta^1:\eta^1:\eta^1:\eta^1$-$P_5)$}$_2${$Cu_6(\mu$-$I)_2(\mu_3$-$I)_4$}]$_n$ (3) 3

2. X-RAY STRUCTURE ANALYSIS 3

2.1 Data Collection and Refinement 3

2.2 Description of crystal structure 2 6

2.3 Selected Bond distances 9

3. SIZE ESTIMATION FROM DOSY 14

3.1 Spectroscopic Details 14

3.2 Size Estimation 14

1. Experimental Part

All reactions were performed under an inert atmosphere of dry nitrogen or argon with standard vacuum, Schlenk, and glove-box techniques. Solvents were purified and degassed by standard procedures. Commercially available chemicals were used without further purification. Complex **1** was prepared as described before.[1]

1.1 Synthesis of $(CH_2Cl_2)_{3.4}$@[{$Cp^{Bn}Fe(\mu_6\text{-}\eta^5:\eta^1:\eta^1:\eta^1:\eta^1:\eta^1\text{-}P_5)$}$_{12}(CuI)_{54}(CH_3CN)_{1.46}$] (2)

Complex **1** (40 mg, 0.055 mmol) was dissolved in CH_2Cl_2 (6 mL) in a long, thin Schlenk tube. On top of the resulting green solution was layered a solution of CuI (63 mg, 0.330 mmol) in a solvent mixture of CH_2Cl_2 (2 mL) and MeCN (3 mL). The reaction mixture immediately turned red at the phase boundary. It was allowed to stand in an undisturbed area at room temperature in the dark for complete diffusion. Within one month dark crystals of **2** were formed. The mother liquor was decanted and the crystals were washed with pentane (3 × 5 mL) and dried under vacuum at room temperature to yield deep-red crystals of **2** (58 mg).

Crystals suitable for X-Ray diffraction analysis were obtained by the following procedure: A solution of CuI (31 mg, 0.16 mmol) in MeCN (3 mL) was carefully layered over a mixture of **1** (40 mg, 0.055 mmol) in toluene. Within one month red multiple twinned crystals were formed. The crystalline material was isolated and washed with hexane (3 × 3 mL). Afterwards, CH_2Cl_2 (6 mL) was added and the mixture was treated in the ultrasonic bath for 20 minutes. The resulting orange-red solution was filtrated, transferred into a thin Schlenk tube and layered with toluene (6 mL). Within three weeks red crystals of **2** were formed.

Analytical data of $(CH_2Cl_2)_{3.4}$@[{$Cp^{Bn}Fe(\mu_6\text{-}\eta^5:\eta^1:\eta^1:\eta^1:\eta^1:\eta^1\text{-}P_5)$}$_{12}(CuI)_{54}(CH_3CN)_{1.46}$] (**2**):

Yield: 58 mg (3.17 µmol, 69 %)

^{1}H NMR (CD_2Cl_2, 400.13 MHz, 300 K): δ [ppm] = 7.0−6.0 (m {br}, 300 H; Ph), 5.0−3.2 (m {br}, 120 H; CH_2), 1.36−1.24 (m {br}, 3 H; CH_3)

^{31}P{^{1}H} NMR (CD_2Cl_2, 161.98 MHz, 300 K): δ [ppm] = 77.3 (s {br}, $\omega_{1/2}$ = 280 Hz)

Positive ion ESI-MS (CH_2Cl_2/CH_3CN): *m/z* (%) = 1897.3 (18) [{$Cp^{Bn}FeP_5$}$_2Cu_3I_2$]$^+$, 1707.4 (39) [{$Cp^{Bn}FeP_5$}$_2Cu_2I$]$^+$, 1587.9 (8) [Cu_9I_8]$^+$, 1515.5 (100) [{$Cp^{Bn}FeP_5$}$_2$Cu]$^+$, 1395.8 (6) [Cu_8I_7]$^+$, 1247.2 (13) [Cu_7I_6(MeCN)]$^+$, 1206.0 (26) [Cu_7I_6]$^+$, 1170.8 (65) [{$Cp^{Bn}FeP_5$}Cu_3I_2]$^+$, 978.9 (35) [{$Cp^{Bn}FeP_5$}Cu_2I]$^+$

Negative ion ESI-MS (CH_2Cl_2/CH_3CN): *m/z* (%) = 2793.6 (0.09) [$Cu_{14}I_{15}$]$^-$, 2603.6 (0.1) [$Cu_{13}I_{14}$]$^-$, 2413.4 (0.21) [$Cu_{12}I_{13}$]$^-$, 2221.6 (0.22) [$Cu_{11}I_{12}$]$^-$, 2031.8 (0.53) [$Cu_{10}I_{11}$]$^-$, 1841.9 (0.84) [Cu_9I_{10}]$^-$, 1649.8 (1.0) [Cu_8I_9]$^-$, 1460.0 (0.8) [Cu_7I_8]$^-$, 1270.1 (1.0) [Cu_6I_7]$^-$, 1078.2 (1.2) [Cu_5I_6]$^-$, 888.3 (2.5) [Cu_4I_5]$^-$, 698.5 (12) [Cu_3I_4]$^-$, 506.6 (35) [Cu_2I_3]$^-$, 316.8 (100) [CuI_2]$^-$

MALDI-TOF (DCTB matrix): *m/z* (%) = 1706 (38) [($Cp^{Bn}FeP_5$)$_2Cu_2I$]$^+$, 1515.0 (100) [($Cp^{Bn}FeP_5$)$_2$Cu]$^+$

IR (KBr): $\tilde{\nu}$ [cm^{-1}] = 3105 (vw; CH), 3085 (w; CH), 3060 (m; CH), 3027 (m; CH), 3003 (vw; CH), 2918 (w; CH_2), 1948 (w), 1881 (w), 1803 (w), 1624 (m), 1603 (s; CC), 1495 (vs; CC), 1454 (s; $\delta(CH_2)$), 1445 (s; $\delta(CH_2)$), 1076 (m), 1030 (m), 732 (vs; δ(Ph)), 696 (vs; δ(Ph)), 520 (w), 489 (m), 462 (w)

Elemental analysis: Calculated (%) for [$(C_{40}H_{35}FeP_5)_{12}(CuI)_{54}(CH_3CN)_{1.46}(CH_2Cl_2)_{3.4}$] (19347 g/mol): C 30.18, H 2.25, Cu 17.74, Fe 3.46, I 35.42, N 0.10, P 9.61; found: C 31.45, H 2.41, Cu 16.7, Fe 4.00, I 34.65, N 0.1, P 9.99.

1.2 Synthesis of [{Cp^{Bn}Fe(μ_5-η^5:η^1:η^1:η^1:η^1-P_5)}$_2${Cu_6(μ-I)$_2$(μ_3-I)$_4$}]$_n$ (3)

A Schlenk tube was charged with a solution of **1** (60 mg, 0.083 mmol) in CH_2Cl_2 (10 mL). Onto this a solution of CuI (47 mg, 0.25 mmol) in CH_3CN (10 mL) was layered. The reaction mixture immediately turned red at the phase boundary and was allowed to stand in an undisturbed area at room temperature for complete diffusion. After one month deep red blocks of **2** and yellow plates of **3** were formed. The yellow plates of **3** were separated mechanically from **2**, washed with CH_2Cl_2 (2 × 4 mL) and pentane (2 × 4 mL) and dried under vacuum at room temperature (25 mg, 35% based on CuI). Analytical data of **3**: Elemental analysis: Calculated (%) for $(C_{40}H_{35}FeP_5)_2(CuI)_6 \cdot CH_2Cl_2$ (2680 g/mol): C 36.29, H 2.71; found: C 36.10, H 2.67.

Analytical data of [{Cp^{Bn}Fe(μ_5-η^5:η^1:η^1:η^1:η^1-P_5)}$_2$(CuI)$_6$]$_n$ (**3**)

Yield: 25 mg (9.33 µmol, 22 %)

Elemental analysis: Calculated (%) for $(C_{40}H_{35}FeP_5)_2(CuI)_6 \cdot CH_2Cl_2$ (2680 g/mol): C 36.29, H 2.71; found: C 36.10, H 2.67.

2. X-Ray Structure Analysis

2.1 Data Collection and Refinement

Crystals of **2** and **3** were taken from a Schlenk flask under a stream of argon and immediately covered with mineral oil (**2**) or perfluorinated Fomblin® mineral oil (**3**) to prevent both decomposition and a loss of solvent. The quickly chosen single crystals covered by a drop of the oil were taken to the pre-centered goniometer head with CryoMount® and directly attached to the diffractometer into a stream of cold nitrogen. X-ray diffraction study of **2** faced many challenges, since the crystals have relatively small size and decompose rapidly losing solvent molecules. Several X-ray diffraction experiments of different single crystals of **2** were performed during the attempts to achieve the best diffraction data. The diffraction power of crystals was very low, and the collection of data at high theta angles required high exposure times.

The diffraction data for structures **2** and **3** were collected at 123 K on an Agilent Technologies SuperNova CCD diffractometer equipped with Atlas detector (**2**) and Gemini R-Ultra diffractometer equipped with Ruby detector (**3**) with CuK_α radiation (λ = 1.54178Å) using ω scans of 1° frames (Table 1S). Absorption corrections were applied analytically from crystal faces using CrysAlisPro software.[2] The structures **2** and **3** were solved by direct methods and refined by full-matrix least-squares method against $|F|^2$ in anisotropic approximation using SHELX97[3] for **3** and SHELX2013[4] programs set for **2**. All non-hydrogen atoms were refined anisotropically. Hydrogen atoms were refined as riding on pivot atoms.

Despite the crystals of **2** contain many heavy atoms, the diffraction pattern fades quickly at d_{hkl}<1 Å. Therefore, only data with $d_{hkl} \geq 0.93$ Å were used in the structure refinement. The structure refinement faced various problems due to low triclinic symmetry resulted in 96 crystallographically independent positions of heavy atoms, severe disorder of Cp^{Bn} ligands accompanied by partial disorder of Cu and Br atoms of inorganic core, and lack of observed high-angle reflections. At the preliminary stages refinement was performed using Konnert-Hendrickson conjugate-gradient algorithm (CGLS instruction in SHELXL97 program), and only final model was refined by full-matrix least-squares method.

Table 1S. Experimental details for compounds 2 and 3

Crystal data	**2**	**3**
Chemical formula	$C_{491.40}H_{438.80}Cl_{6.80}Cu_{54}Fe_{12}I_{54}N_4P_{60}$	$C_{40}H_{35}Cu_3FeI_3P_5\cdot0.5(CH_2Cl_2)$
M_r	19453.26	1340.16
Crystal system, space group	Triclinic, $P\bar{1}$	Triclinic, $P\bar{1}$
Temperature (K)	123.0	123.0
a, b, c (Å)	27.7249(8), 27.7730(7), 27.9764(8)	10.2110(8), 10.8762(8), 19.4787(12)
α, β, γ (°)	119.783(3), 105.310(2), 96.155(2)	96.601(6), 102.067(6), 92.147(6)
V (Å^3)	17286.3(8)	2097.2 (3)
Z	1	2
F(000)	9171	1286
Radiation type	Cu $K\alpha$	Cu $K\alpha$
D_x (g·sm^{-3})	1.869	2.122
μ (mm^{-1})	24.468	24.40
Crystal shape	prism	plate
Colour	red-brown	yellow-orange
Crystal size (mm)	0.06 × 0.06 × 0.11	0.45 × 0.35 × 0.02
Data collection		
Diffractometer	Agilent Technologies SuperNova diffractometer, Atlas detector	Agilent Technologies Gemini R-Ultra diffractometer, Ruby detector
Absorption correction	multi-scan,<br>CrysAlisPro, Oxford Diffraction Ltd., Version 1.171.33.61 (release 04-03-2010 CrysAlis171 .NET)<br>(compiled Mar 4 2010,15:49:12) Empirical absorption correction using spherical harmonics, implemented in SCALE3 ABSPACK scaling algorithm.	Analytical<br>*CrysAlis PRO*, Oxford Diffraction Ltd., Version 1.171.33.41 (release 06-05-2009 CrysAlis171 .NET) (compiled May 6 2009,17:20:42) Analytical numeric absorption correction using a multifaceted crystal model based on expressions derived by R.C. Clark & J.S. Reid. (Clark, R. C. & Reid, J. S. (1995). Acta Cryst. A51, 887-897)
T_{min}, T_{max}	0.609, 1.000	0.028, 0.620
No. of measured, independent and observed [$I > 2\sigma(I)$] reflections	103712, 44589, 20080	13889, 7204, 5463
R_{int}	0.064	0.083
$(\sin \theta/\lambda)_{max}$ (Å^{-1})	0.540	0.597
Range of h, k, l	h = -29→27, k = -29→28, l = -29→15	h = -11→12, k = -10→12, l = -23→22
Refinement		
$R[F^2 > 2\sigma(F^2)]$, $wR(F^2)$, S	0.0884, 0.2811, 0.938	0.065, 0.171, 0.97
No. of reflections	44589	7204
No. of parameters	1661	487
No. of restraints	226	0
H-atom treatment	-	H-atom parameters constrained
Weighting scheme	$w = 1/[\sigma^2(F_o^2) + (0.1639P)^2]$<br>where $P = (F_o^2 + 2F_c^2)/3$	$w = 1/[\sigma^2(F_o^2) + (0.1214P)^2]$<br>where $P = (F_o^2 + 2F_c^2)/3$
$\Delta\rangle_{max}$, $\Delta\rangle_{min}$ (e Å^{-3})	1.842, -1.584	2.95, -2.05

Computer programs for **2**: *CrysAlis PRO*, Agilent Technologies, Version 1.171.33.61 (release 04-03-2010 CrysAlis171.NET) (compiled Mar 4 2010, 15:49:12), *SHELXS97* (Sheldrick, 1998), *SHELXL2013* (Sheldrick, 2013).

Computer programs for **3**: *CrysAlis PRO*, Agilent Technologies, Version 1.171.33.41 (release 06-05-2009 CrysAlis171.NET) (compiled May 6 2009, 17:20:42), *SHELXS97* (Sheldrick, 1990), *SHELXL97* (Sheldrick, 1997).

The occupancies for partly vacant Cu and I positions were determined after refinement with their isotropic displacement parameters fixed at U_{iso} of 0.06 Å^{-1} that is approximately equal to U_{iso} of fully occupied Cu, I and Fe atoms. The resulting occupancies were then fixed, and conventional refinement procedure with refining displacement parameters was performed in iso- and then in anisotropic approximation. Some positions of heavy atoms were split as a result of unreasonably elongated a.d.p. ellipsoids and relatively high surrounding residual density. Final occupancy factors for iodine atoms were set using information on chemical composition, crystallochemical and charge balance requirements. The carbon atoms of Cp rings belonging to a $Cp(CH_2Ph)_5$

substituents were refined anisotropically using ISOR instructions of SHELX. The positions of severely disordered CH_2Ph fragments and NCMe ligands were located from $\Delta\rho$ maps and refined in the rigid body approximation with U_{iso} at 0.15 $Å^{-1}$ level as a single position to reduce the number of refined parameters. The hydrogen atoms were not set in calculated positions, because of uncertain positions of the pivot carbon atoms. Some of the coordinated MeCN molecules, which are to complete Cu ions environment, were not located from electron density map due to their disorder.

The guest molecules of CH_2Cl_2 in the inner cavity of supramolecules are disordered over 6 positions, note that only three of them are crystallographically independent. The refinement of occupancies for Cl atoms gave sum occupancy of 3.4 CH_2Cl_2 molecules per supramolecule. Due to uncertainty of the position of C atom the C-Cl distances were restrained with SADI instructions.

A few solvent molecules were located from residual electron density map; however, the solvent portion of the compound **2** cannot be even approximately estimated from the crystal structure. The crystal structure contains voids in the packing of supermolecules of ~3619 $Å^3$ per unit cell, in which single electron density peaks are only found that however do not allow localization of any solvent molecule. The estimation based on residual electron density made with SQUEEZE/PLATON allows assigning the non-localized solvent portion as 16 C_7H_8, 17 CH_2Cl_2 or 37 MeCN per unit cell.

The structural formula for the crystal structure **3** derived from the structural data is therefore $(CH_2Cl_2)_{3.4}$@$[(CuI)_{54}((C_{40}H_{35})FeP_5)_{12}(CH_3CN)_{1.46}]\cdot 2.54(CH_3CN)\cdot 0.8C_7H_8$ without taking into account non-localized solvent molecules.

Packing motifs were analyzed with TOPOS 4.0 Professional program suit for crystal chemical analysis.[5] Bond lengths and bond angles are summarized in Tables 2S and 3S. CCDC-1008152 (compound **2**), -1008153 (compound **3**) contain the supplementary crystallographic data for this publication. These data can be obtained free of charge at www.ccdc.cam.ac.uk/conts/retrieving.html (or from the Cambridge Crystallographic Data Centre, 12 Union Road, Cambridge CB2 1EZ, UK; Fax: + 44-1223-336-033; e-mail: deposit@ccdc.cam.ac.uk).

2.2 Description of crystal structure 2

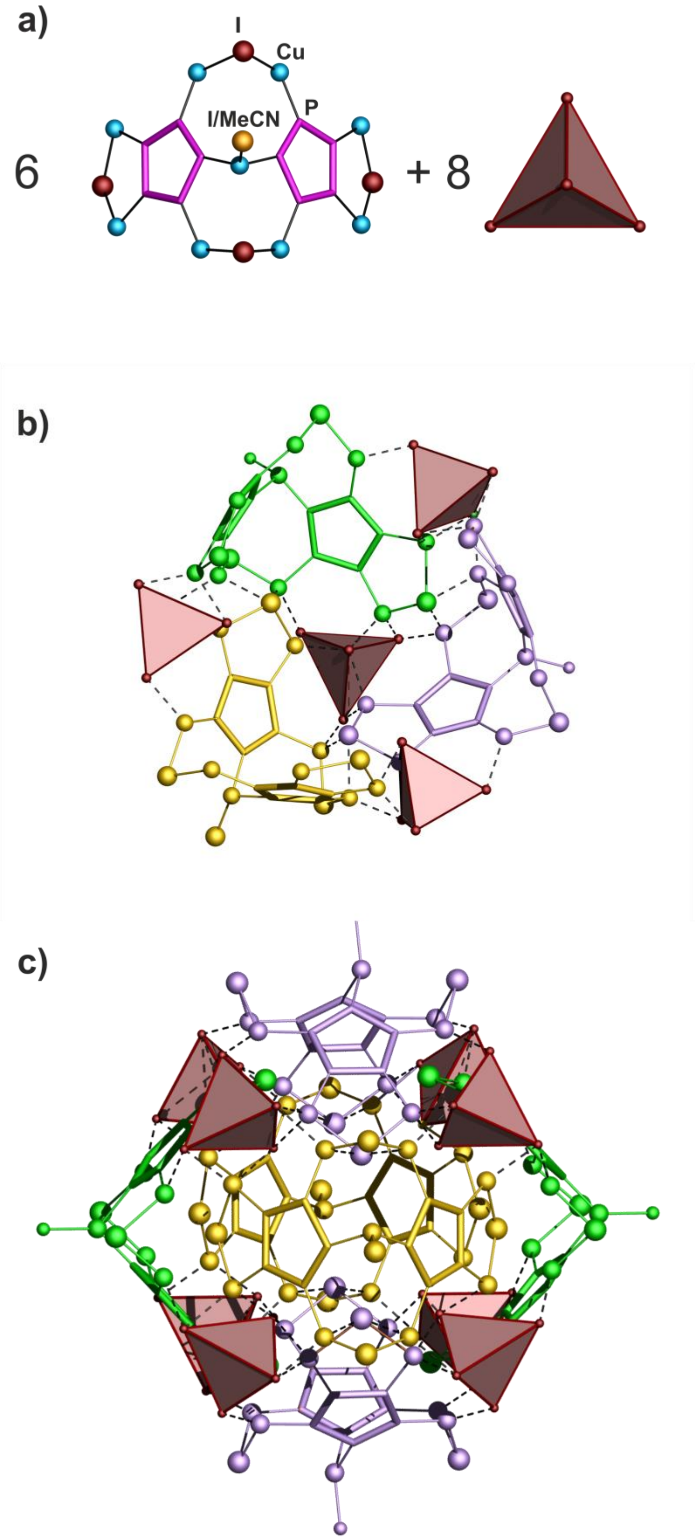

Figure 1S. The stepwise construction of the idealized scaffold (without taking into account partly occupied positions) of **2**. A combination of 6 $(Cp^{Bn}FeP_5)_2(CuX)(Cu_2I)_4$, (X = I, MeCN) fragments and 8 CuI_4 tetrahedrons (a) gives half-shell (b) and the idealized scaffold $(Cp^{Bn}FeP_5)_{12}Cu_{62}I_{58}(MeCN)_4$ (c).

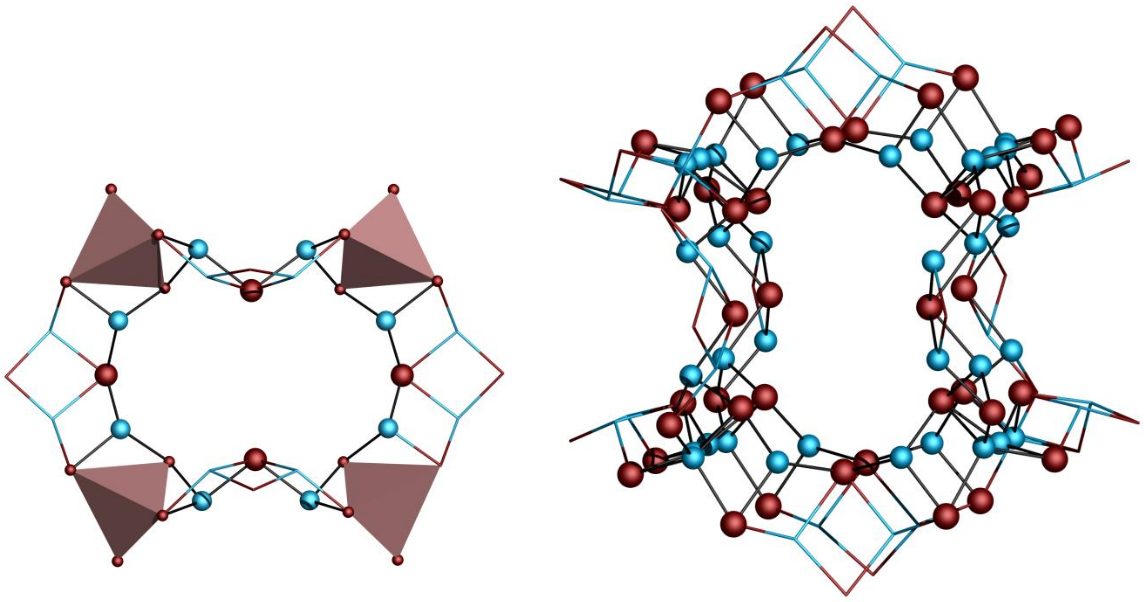

Figure 2S. The way of bonding of $\{CuI_4\}$ tetrahedra and $\{Cu_4I_2\}$ units giving the $\{CuI\}_{56}$ framework in idealized inorganic scaffold in **2** (left) and its fully occupied part comprising 34 copper and 40 iodine ions (right). The disordered positions of Cu and I are shown as sticks.

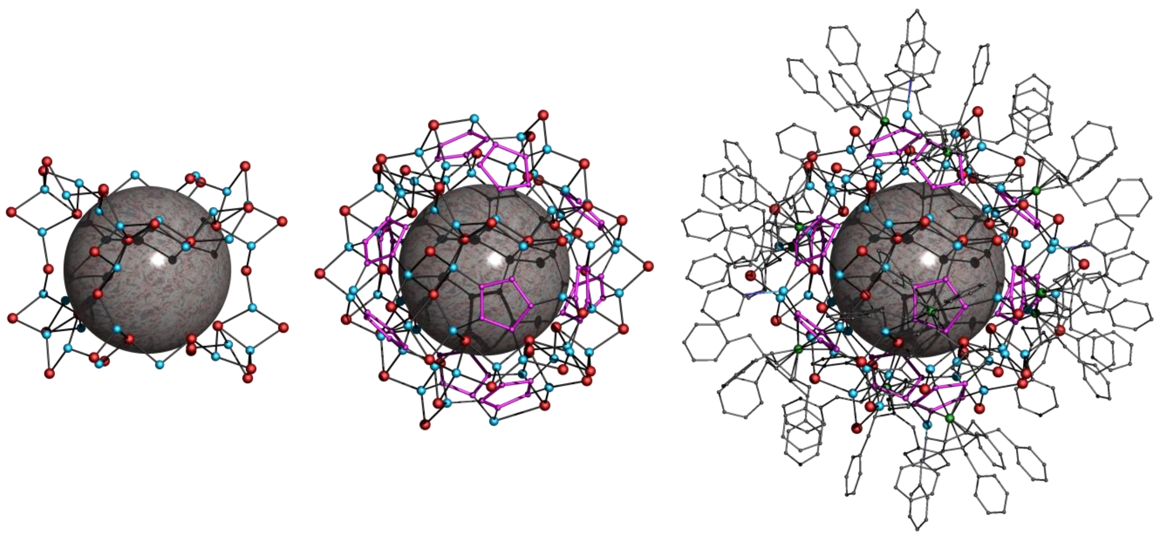

Figure 3S. *left*: The irreducible scaffold of **2**. *middle:* The idealized scaffold of **2.** *right*: One of the possible supramolecules in **2**. Hydrogen atoms are omitted for clarity.

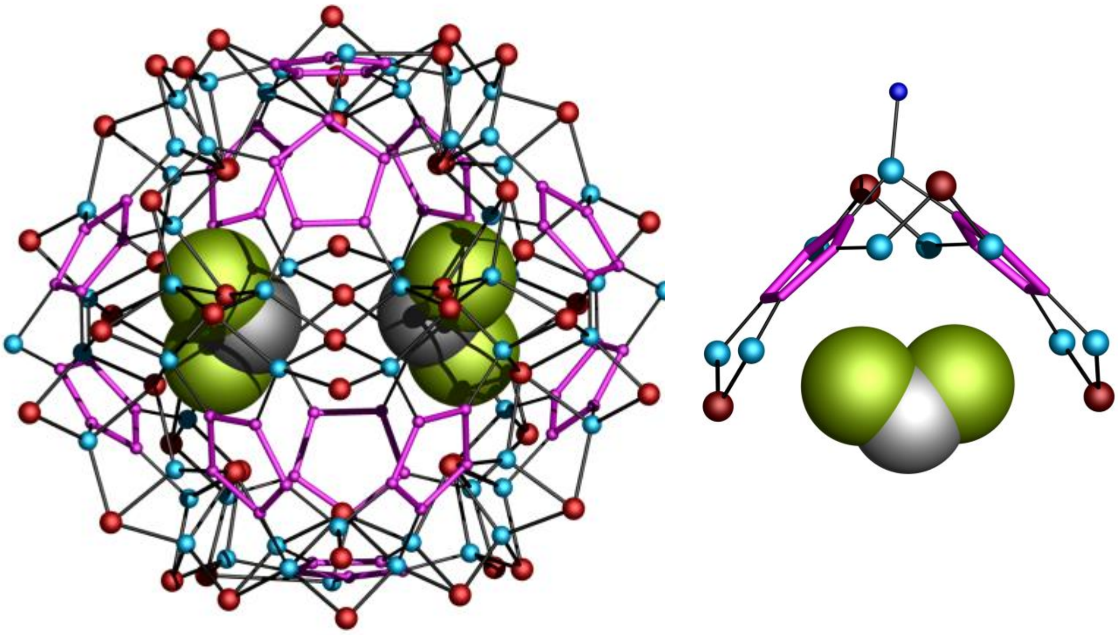

a **b**

Figure 4S. *left:* The arrangement of two guest CH_2Cl_2 molecules in the cavity of supramolecule **2**. *right:* The complementarity of a guest molecule to one of the $(Cp^{Bn}FeP_5)_2(CuMeCN)(Cu_2I)_4$ building block of **2**.

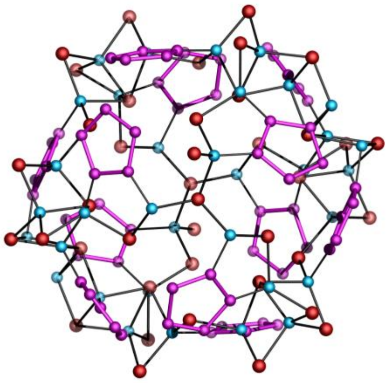

Figure 5S: Fully occupied part of the scaffold with coordinated *cyclo*-P_5 ligands of **2.**

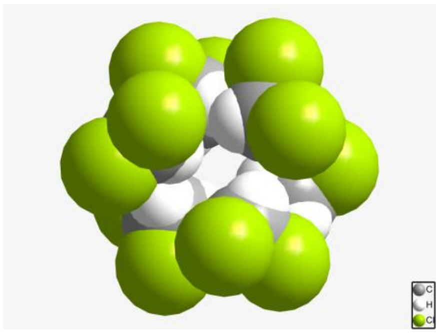

Figure 6S: Arrangement of 6 non-contradictory positions of guest CH_2Cl_2 molecules inside the cavity.

2.3 Selected Bond distances

Table 2S. Selected bond lengths (Å, °) for compound 2

**Cu-P**		**Cu-I**	
Cu1A—P34	2.168 (18)	Cu1A—I5B^{i}	2.95 (2)
Cu1A—P54	2.281 (18)	Cu2A—I7B	2.931 (18)
Cu1B—P34	2.16 (3)	Cu3A—I3B	2.548 (11)
Cu1B—P54	2.23 (2)	Cu3A—I3A	2.615 (14)
Cu2A—P62	2.109 (17)	Cu3A—I3C	2.631 (4)
Cu2A—P43	2.335 (18)	Cu3A—I16	2.792 (4)
Cu2B—P62	2.16 (4)	Cu3B—I3B^{i}	2.515 (11)
Cu2B—P43	2.29 (4)	Cu3B—I25	2.644 (5)
Cu3A—P35	2.251 (9)	Cu3B—I3A^{i}	2.734 (14)
Cu3B—P53^{i}	2.226 (6)	Cu3B—I16^{i}	2.768 (4)
Cu4A—P24	2.259 (8)	Cu3C—I21	2.664 (5)
Cu5A—P55^{i}	2.220 (8)	Cu3C—I3C	2.676 (4)
Cu5B—P33	2.232 (8)	Cu3C—I12	2.683 (3)
Cu6A—P63^{i}	2.237 (8)	Cu3C—I4C	2.689 (5)
Cu6B—P44^{i}	2.257 (9)	I3A—Cu3B^{i}	2.734 (14)
Cu7A—P42	2.211 (8)	I3B—Cu3B^{i}	2.515 (11)
Cu7B—P61	2.244 (8)	I3C—Cu3	2.585 (3)
Cu8A—P15	2.19 (5)	Cu4A—I4B	2.411 (13)
Cu8A—P25	2.22 (4)	Cu4A—I28	2.641 (5)
Cu1—P21	2.242 (8)	Cu4A—I4A	2.748 (13)
Cu2—P11	2.240 (7)	Cu4A—I20	2.826 (5)
Cu3—P41	2.253 (8)	Cu4B—P14	2.259 (8)
Cu11—P12	2.255 (8)	Cu4B—I4A	2.412 (19)
Cu12—P13	2.259 (7)	Cu4B—I4C	2.646 (5)
Cu13—P52^{i}	2.242 (8)	Cu4B—I4B	2.690 (15)
Cu14—P23	2.241 (7)	Cu4B—I20	2.803 (4)
Cu18—P32	2.274 (8)	I4C—Cu19	2.583 (3)
Cu19—P31	2.279 (8)	Cu5A—I5A	2.532 (6)
Cu20—P65	2.245 (7)	Cu5A—I24	2.689 (5)
Cu21—P22	2.261 (7)	Cu5A—I23	2.733 (5)
Cu22—P51^{i}	2.253 (7)	Cu5A—I5B	2.932 (12)
Cu23—P45^{i}	2.239 (7)	Cu5B—I5A^{i}	2.564 (6)
Cu24—P64^{i}	2.256 (7)	Cu5B—I30	2.630 (5)
P44—Cu6B^{i}	2.257 (9)	Cu5B—I5B^{i}	2.641 (10)
P45—Cu23^{i}	2.239 (7)	Cu5B—I23^{i}	2.799 (5)
P51—Cu22^{i}	2.253 (7)	I5A—Cu5B^{i}	2.564 (6)
P52—Cu13^{i}	2.243 (8)	I5B—Cu5B^{i}	2.641 (10)
P53—Cu3B^{i}	2.226 (6)	I5B—Cu1A^{i}	2.95 (2)
P55—Cu5A^{i}	2.220 (8)	Cu6A—I6A	2.577 (19)
P63—Cu6A^{i}	2.237 (8)	Cu6A—I27	2.612 (5)

P64—Cu24^{i}	2.256 (7)	Cu6A—I6B	2.553 (17)
**Fe-P**		Cu6A—I22	2.811 (4)
Fe1—P11	2.370 (6)	Cu6B—I6A	2.611 (18)
Fe1—P12	2.390 (7)	Cu6B—I6B	2.566 (18)
Fe1—P13	2.377 (7)	Cu6B—I29	2.650 (5)
Fe1—P14	2.363 (8)	Cu6B—I22	2.753 (4)
Fe1—P15	2.385 (7)	Cu6B—Cu21	2.891 (5)
Fe2—P25	2.373 (8)	Cu7A—I7A	2.499 (6)
Fe2—P24	2.376 (8)	Cu7A—I21	2.638 (5)
Fe2—P22	2.376 (8)	Cu7A—I19	2.761 (4)
Fe2—P21	2.378 (8)	Cu7A—I7B	2.891 (8)
Fe2—P23	2.393 (8)	Cu7B—I7A	2.555 (5)
Fe3—P31	2.345 (8)	Cu7B—I18	2.625 (4)
Fe3—P32	2.364 (7)	Cu7B—I7B	2.642 (6)
Fe3—P35	2.380 (9)	Cu7B—I19	2.781 (4)
Fe3—P34	2.398 (9)	Cu8A—I8A	2.41 (5)
Fe3—P33	2.404 (7)	I9A—Cu2	2.41 (2)
Fe4—P44	2.357 (8)	I9A—Cu1	2.59 (3)
Fe4—P43	2.357 (8)	I9B—Cu1	2.56 (2)
Fe4—P45	2.378 (6)	I9B—Cu2	2.675 (17)
Fe4—P41	2.379 (7)	Cu1—I26	2.640 (4)
Fe4—P42	2.382 (7)	Cu1—I13	2.788 (4)
Fe5—P55	2.353 (8)	Cu2—I17	2.635 (4)
Fe5—P51	2.366 (6)	Cu2—I13	2.800 (3)
Fe5—P53	2.373 (6)	Cu3—I12	2.683 (4)
Fe5—P54	2.374 (8)	Cu3—I16	2.683 (3)
Fe5—P52	2.389 (7)	Cu11—I18	2.595 (3)
Fe6—P65	2.372 (7)	Cu11—I19	2.683 (3)
Fe6—P62	2.373 (9)	Cu11—I11	2.688 (3)
Fe6—P61	2.376 (9)	Cu12—I21	2.586 (3)
Fe6—P64	2.383 (7)	Cu12—I12	2.681 (3)
Fe6—P63	2.391 (8)	Cu12—I19	2.682 (4)
**Fe-C**		Cu13—I26	2.583 (4)
Fe1—C14	2.06 (2)	Cu13—I13	2.681 (3)
Fe1—C11	2.06 (2)	Cu13—I14	2.683 (3)
Fe1—C12	2.06 (3)	Cu14—I27	2.586 (4)
Fe1—C15	2.06 (2)	Cu14—I15	2.674 (4)
Fe1—C13	2.12 (2)	Cu14—I22	2.679 (4)
Fe2—C25	2.060 (18)	Cu15—I25	2.673 (4)
Fe2—C21	2.065 (19)	Cu15—I29	2.675 (4)
Fe2—C24	2.082 (18)	Cu15—I26	2.675 (4)
Fe2—C22	2.09 (2)	Cu15—I14	2.683 (4)
Fe2—C23	2.101 (18)	Cu16—I24	2.665 (4)
Fe3—C34	2.061 (19)	Cu16—I18	2.666 (3)

Fe3—C33	2.069 (17)	Cu16—I17	2.672 (4)
Fe3—C35	2.074 (17)	Cu16—I11	2.687 (3)
Fe3—C32	2.088 (17)	Cu17—I15	2.666 (4)
Fe3—C31	2.091 (17)	Cu17—I27	2.674 (4)
Fe4—C41	2.05 (2)	Cu17—I28	2.678 (4)
Fe4—C43	2.09 (3)	Cu17—I30	2.683 (5)
Fe4—C44	2.08 (3)	Cu18—I28	2.584 (4)
Fe4—C45	2.18 (3)	Cu18—I15	2.673 (4)
Fe4—C42	2.22 (2)	Cu18—I20	2.680 (4)
Fe5—C53	2.05 (2)	Cu19—I12	2.668 (4)
Fe5—C55	2.05 (2)	Cu19—I20	2.701 (4)
Fe5—C52	2.08 (2)	Cu20—I24	2.577 (4)
Fe5—C54	2.08 (2)	Cu20—I11	2.688 (4)
Fe5—C51	2.09 (2)	Cu20—I23	2.694 (3)
Fe6—C61	2.01 (3)	Cu21—I29	2.582 (3)
Fe6—C62	2.03 (3)	Cu21—I14	2.667 (4)
Fe6—C64	2.05 (3)	Cu21—I22	2.697 (4)
Fe6—C63	2.08 (3)	Cu22—I17	2.590 (3)
Fe6—C65	2.09 (3)	Cu22—I11	2.676 (3)
**P-P**		Cu22—I13	2.680 (4)
P11—P15	2.096 (10)	Cu23—I25	2.579 (3)
P11—P12	2.100 (9)	Cu23—I14	2.684 (3)
P12—P13	2.090 (9)	Cu23—I16^{i}	2.690 (4)
P13—P14	2.077 (10)	Cu24—I30	2.586 (4)
P14—P15	2.097 (10)	Cu24—I23^{i}	2.678 (4)
P21—P22	2.089 (9)	Cu24—I15	2.684 (4)
P21—P25	2.118 (10)	I16—Cu23^{i}	2.689 (4)
P22—P23	2.106 (10)	I16—Cu3B^{i}	2.768 (4)
P23—P24	2.090 (9)	I23—Cu24^{i}	2.678 (4)
P24—P25	2.075 (11)	I23—Cu5B^{i}	2.799 (5)
P31—P35	2.071 (10)		
P31—P32	2.083 (10)		
P32—P33	2.076 (10)		
P33—P34	2.123 (11)		
P34—P35	2.100 (10)		
P41—P42	2.082 (9)		
P41—P45	2.110 (9)		
P42—P43	2.106 (11)		
P43—P44	2.093 (10)		
P44—P45	2.091 (10)		
P51—P55	2.084 (10)		
P51—P52	2.103 (8)		
P52—P53	2.111 (9)		
P53—P54	2.095 (11)		

P54—P55	2.080 (9)		
P61—P65	2.082 (9)		
P61—P62	2.097 (10)		
P62—P63	2.098 (10)		
P63—P64	2.097 (9)		
P64—P65	2.097 (10)		

Table 3S. Selected geometric parameters (Å, º) for compound 3

Cu1—P1	2.301 (3)	I3—Cu3^{iii}	2.6816 (15)
Cu1—P5^{ii}	2.268 (3)	Fe1—P1	2.363 (3)
Cu2—P2	2.266 (3)	Fe1—P2	2.426 (3)
Cu3—P3	2.262 (3)	Fe1—P3	2.372 (3)
Cu1—Cu2^{i}	3.0013 (19)	Fe1—P4	2.386 (2)
Cu2—Cu1^{i}	3.0013 (19)	Fe1—P5	2.391 (3)
Cu1—I1^{i}	2.6452 (14)	Fe1—C1	2.122 (10)
Cu1—I2	2.5966 (15)	Fe1—C2	2.090 (10)
Cu2—I1	2.7920 (17)	Fe1—C3	2.099 (9)
Cu2—I2^{i}	2.5765 (14)	Fe1—C4	2.105 (9)
Cu2—I3^{iii}	2.6537 (16)	Fe1—C5	2.135 (9)
Cu3—I3	2.6690 (16)	P1—P2	2.102 (3)
Cu3—I3^{iii}	2.6816 (15)	P1—P5	2.115 (3)
Cu3—I1^{iii}	2.5884 (15)	P2—P3	2.099 (3)
I1—Cu1^{i}	2.6453 (14)	P3—P4	2.117 (3)
I1—Cu3^{iii}	2.5885 (15)	P4—P5	2.102 (3)
I2—Cu2^{i}	2.5764 (14)	P5—Cu1^{ii}	2.268 (3)
I3—Cu2^{iii}	2.6536 (16)		
I1^{i}—Cu1—Cu2^{i}	58.87 (4)	P2—Cu2—I1	115.21 (9)
I2—Cu1—Cu2^{i}	54.22 (4)	P2—Cu2—I2^{i}	114.35 (8)
I2—Cu1—I1^{i}	109.73 (5)	P2—Cu2—I3^{iii}	105.86 (8)
P1—Cu1—Cu2^{i}	129.01 (8)	I1^{iii}—Cu3—I3	103.56 (5)
P1—Cu1—I1^{i}	109.45 (8)	I1^{iii}—Cu3—I3^{iii}	107.93 (5)
P1—Cu1—I2	96.68 (8)	I3—Cu3—I3^{iii}	103.97 (5)
P5^{ii}—Cu1—Cu2^{i}	121.19 (8)	P3—Cu3—I1^{iii}	126.85 (9)
P5^{ii}—Cu1—I1^{i}	112.19 (8)	P3—Cu3—I3	102.91 (9)
P5^{ii}—Cu1—I2	118.26 (9)	P3—Cu3—I3^{iii}	109.12 (8)
P5^{ii}—Cu1—P1	109.27 (10)	Cu1^{i}—I1—Cu2	66.94 (4)
I1—Cu2—Cu1^{i}	54.19 (4)	Cu3^{iii}—I1—Cu1^{i}	109.70 (5)
I2^{i}—Cu2—Cu1^{i}	54.85 (4)	Cu3^{iii}—I1—Cu2	75.45 (5)
I2^{i}—Cu2—I1	105.91 (5)	Cu2^{i}—I2—Cu1	70.92 (5)
I2^{i}—Cu2—I3^{iii}	116.11 (6)	Cu2^{iii}—I3—Cu3	76.52 (5)
I3^{iii}—Cu2—Cu1^{i}	104.03 (5)	Cu2^{iii}—I3—Cu3^{iii}	90.03 (5)
I3^{iii}—Cu2—I1	98.65 (5)	Cu3—I3—Cu3^{iii}	76.03 (5)
P2—Cu2—Cu1^{i}	149.63 (9)		

Symmetry code(s): (i) -*x*+1, -*y*+2, -*z*; (ii) -*x*, -*y*+2, -*z*; (iii) -*x*+1, -*y*+1, -*z*.

3. Size Estimation from DOSY

3.1 Spectroscopic Details

The DOSY spectra were recorded on an Avance III HD 600 (600.13 MHz) spectrometer equipped with a z-gradient (53.5 Gauss/cm), 5 mm triple resonance CPP-BBO and BCU II unit at 300 K and on a Avance III 600 (600.25 MHz) spectrometer equipped with a z-gradient (53.5 Gauss/cm), 5 mm TCI cryo probe and BVT 3000 unit at 298 K. The NMR data was processed with the Bruker program TopSpin® 3.2 and the diffusion coefficient was calculated with the Bruker software *T1/T2* relaxation package.

For the calibration of the ^{1}H chemical shifts and for the temperature- and viscosity-correction of the diffusion coefficients, TMS (tetramethylsilane) was added. The ^{1}H-diffusion measurement was performed with the convection suppressing DSTE (double stimulated echo) pulse sequence, developed by Mueller and Jerschow[6a] in a pseudo 2D mode. 120 dummy scans and 16 scans were used with a relaxation delay of 2 s. Sinusoidal shapes were used for the gradient and a linear gradient ramp with 20 increments between 5 and 95 % of the maximum gradient strength was applied for the diffusion relevant gradients. For the homospoil gradients, -13.7, 20 and -17.13 G cm^{-1} were applied. The length of the gradient pulse δ was adjusted for every species in the sample to achieve appropriate signal attenuation curves, giving values for δ of 2.0 ms for TMS and 3.7 ms for the supramolecule. A diffusion time Δ of 50 ms was used.

3.2 Size Estimation

From crystallographic data the diameter of the **2** cluster was estimated to be d = 37.0 Å (r = 18.5 Å) from the maximum H-H distance plus twice the Van-der-Waals radius of a proton (1.2 Å[7]).

From diffusion-ordered spectroscopy (DOSY) experiments[6b-c] the translational self-diffusion coefficient D of molecules in solution can be calculated according to the Stejskal-Tanner equation.[6] With the diffusion coefficients D of the analyte and of TMS (acting as viscosity reference), the hydrodynamic radius r_H of the analyte can be estimated following the Stokes-Einstein equation[8] to be r_H = 20.72 Å.

This in very good agreement with the crystal-derived radius of 18.5 Å in the solid state. Therefore these results clearly indicate the presence of the intact supramolecule in solution.

[1] F. Dielmann, R. Merkle, S. Heinl, M. Scheer, *Zeitschrift für Naturforschung* **2009**, *64*, 3-10.
[2] *CrysAlis PRO*, Agilent Technologies, 2006-2014.
[3] G. M. Sheldrick, *Acta Cryst.* **2008**, *A64*, 112-122.
[4] G. M. Sheldrick. SHELX2013, University of Göttingen, 2013.
[5] V.A. Blatov, *Cryst. Comp. Newsletter*, **2006**, *7*, 4-38 (http://www.iucr.org/iucr-top/comm/ccom/newsletters/).
[6] a) A. Jerschow, N. Müller, *J. Magn. Reson.* **1997**, *125*, 372-375. b) C. S. Johnson, *Prog. Nucl. Magn. Reson. Spectrosc.* **1999**, *34*, 203-256; c) W. S. Price, *Concepts Magn. Reson.* **1998**, *10*, 197-237; d) E. O. Stejskal, J. E. Tanner, *The Journal of Chemical Physics* **1965**, *42*, 288-292.
[7] M. Scheer, A. Schindler, C. Groeger, A. V. Virovets, E. V. Peresypkina, *Angew. Chem., Int. Ed.* **2009**, *48*, 5046-5049.
[8] A. Macchioni, G. Ciancaleoni, C. Zuccaccia, D. Zuccaccia, *Chem. Soc. Rev.* **2008**, *37*, 479-489.
